# (μ-2,2′-Bipyrimidine-κ^4^ *N* ^1^,*N* ^1′^:*N* ^3^,*N* ^3′^)bis­[triaqua­(sulfato-κ*O*)manganese(II)]

**DOI:** 10.1107/S160053681103604X

**Published:** 2011-09-14

**Authors:** Kwang Ha

**Affiliations:** a School of Applied Chemical Engineering, The Research Institute of Catalysis, Chonnam National University, Gwangju 500-757, Republic of Korea

## Abstract

The title complex, [Mn_2_(SO_4_)_2_(C_8_H_6_N_4_)(H_2_O)_6_], is the second monoclinic polymorph [De Munno *et al.* (1995 ▶). *Inorg. Chem.* **34**, 408–411; Hong *et al.* (1996 ▶). *Polyhedron*, **15**, 447–452]. The asymmetric unit contains two crystallographically independent half-mol­ecules of the binuclear Mn^II^ complex; an inversion centre is located at the centroid of each complex. The two Mn^II^ atoms in each complex mol­ecules are bridged by a bis-chelating 2,2′-bipyrimidine (bpym) ligand and each Mn^II^ atom is six-coordinated in a considerably distorted octa­hedral environment defined by two N atoms of the bridging bpym ligand and four O atoms from one sulfato anionic ligand and three water mol­ecules. In the crystal, the complex mol­ecules are linked by O—H⋯O hydrogen bonds between the water and sulfato ligands, forming a three-dimensional network.

## Related literature

For the crystal structure of the title complex in the same space group but with different cell parameters, see: De Munno *et al.* (1995 ▶); Hong *et al.* (1996 ▶). For the synthesis and crystal structure of [Mn_2_(H_2_O)_8_(bpym)](SO_4_)_2_·2H_2_O, see: Ha (2011 ▶).
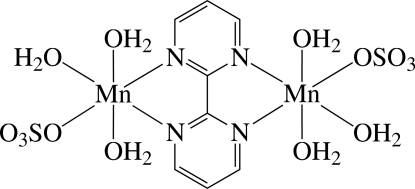


## Experimental

### 

#### Crystal data

[Mn_2_(SO_4_)_2_(C_8_H_6_N_4_)(H_2_O)_6_]
*M* *_r_* = 568.26
Monoclinic, 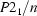

*a* = 12.4401 (18) Å
*b* = 13.2640 (19) Å
*c* = 12.8951 (18) Å
β = 117.199 (3)°
*V* = 1892.5 (5) Å^3^
*Z* = 4
Mo *K*α radiation
μ = 1.64 mm^−1^
*T* = 200 K
0.33 × 0.23 × 0.20 mm

#### Data collection

Bruker SMART 1000 CCD diffractometer
Absorption correction: multi-scan (*SADABS*; Bruker, 2000 ▶) *T*_min_ = 0.505, *T*_max_ = 0.721
13624 measured reflections
4652 independent reflections
3069 reflections with *I* > 2σ(*I*)
*R*_int_ = 0.042

#### Refinement

*R*[*F* ^2^ > 2σ(*F* ^2^)] = 0.038
*wR*(*F* ^2^) = 0.110
*S* = 1.08
4652 reflections
272 parameters
H-atom parameters constrained
Δρ_max_ = 0.75 e Å^−3^
Δρ_min_ = −0.62 e Å^−3^

### 

Data collection: *SMART* (Bruker, 2000 ▶); cell refinement: *SAINT* (Bruker, 2000 ▶); data reduction: *SAINT*; program(s) used to solve structure: *SHELXS97* (Sheldrick, 2008 ▶); program(s) used to refine structure: *SHELXL97* (Sheldrick, 2008 ▶); molecular graphics: *ORTEP-3* (Farrugia, 1997 ▶) and *PLATON* (Spek, 2009 ▶); software used to prepare material for publication: *SHELXL97*.

## Supplementary Material

Crystal structure: contains datablock(s) global, I. DOI: 10.1107/S160053681103604X/is2772sup1.cif

Structure factors: contains datablock(s) I. DOI: 10.1107/S160053681103604X/is2772Isup2.hkl

Additional supplementary materials:  crystallographic information; 3D view; checkCIF report

## Figures and Tables

**Table 1:** Selected bond lengths (Å)

Mn1—O4	2.103 (2)
Mn1—O2	2.1295 (19)
Mn1—O1	2.172 (2)
Mn1—O3	2.190 (2)
Mn1—N1	2.303 (2)
Mn1—N2	2.308 (2)
Mn2—O11	2.105 (2)
Mn2—O9	2.1327 (19)
Mn2—O8	2.181 (2)
Mn2—O10	2.184 (2)
Mn2—N3	2.287 (2)
Mn2—N4	2.332 (2)

**Table 2:** Hydrogen-bond geometry (Å, °)

*D*—H⋯*A*	*D*—H	H⋯*A*	*D*⋯*A*	*D*—H⋯*A*
O1—H1*A*⋯O6^i^	0.84	1.88	2.709 (3)	170
O1—H1*B*⋯O12^ii^	0.84	1.90	2.700 (3)	160
O2—H2*A*⋯O13^iii^	0.84	1.86	2.655 (3)	158
O2—H2*B*⋯O14^i^	0.84	1.98	2.804 (3)	168
O3—H3*A*⋯O12^iii^	0.84	2.60	3.434 (4)	175
O3—H3*B*⋯O14^iv^	0.84	1.93	2.721 (3)	157
O8—H8*A*⋯O13^v^	0.84	1.91	2.745 (3)	177
O8—H8*B*⋯O5^ii^	0.84	1.93	2.766 (3)	173
O9—H9*A*⋯O6	0.84	1.80	2.636 (3)	178
O9—H9*B*⋯O4^i^	0.84	2.06	2.839 (3)	153
O10—H10*A*⋯O5	0.84	1.98	2.804 (3)	165
O10—H10*B*⋯O7^vi^	0.84	1.87	2.705 (3)	174

Symmetry codes: (i) 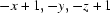; (ii) 
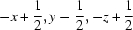
; (iii) 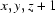; (iv) 
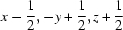
; (v) 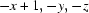; (vi) 
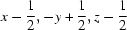
.

**Fig. 1.**
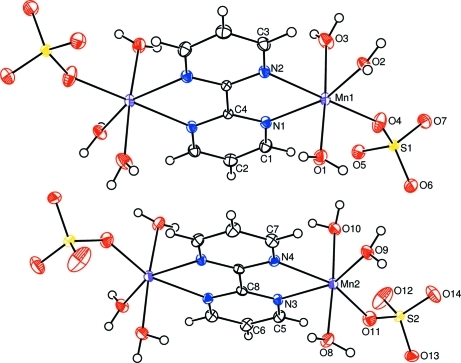
The structure of the title complex, with displacement ellipsoids drawn at the 30% probability level for non-H atoms; H atoms are shown as small circles of arbitrary radius. Unlabelled atoms are generated by the application of the inversion centres.

**Fig. 2.**
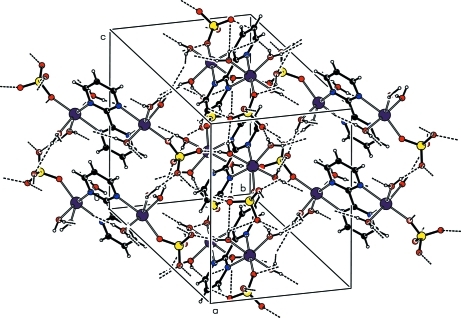
View of the unit-cell contents of the title complex. Hydrogen-bond interactions are drawn with dashed lines.

## supplementary crystallographic information

### Comment

The asymmetric unit of the title complex, [Mn_2_(SO_4_)_2_(H_2_O)_6_(bpym)] (where bpym is 2,2'-bipyrimidine, C_8_H_6_N_4_), contains two crystallographically independent half-molecules of the dinuclear Mn^II^ complex; an inversion centre is located at the centroid of each complex (Fig. 1). The two complexes are chemically identical, but somewhat different in geometry. The crystal structures of the complex were previously reported in the same space group *P*2_1_/*n* (De Munno *et al.*, 1995; Hong *et al.*, 1996). The structure presented here is essentially the same as the published, however, the components of a unit cell and the cell parameters are quite different. Each asymmetric unit of the reported structures contains one half-molecule of the dinuclear complex.

In both complexes, two Mn^II^ ions are bridged by a bis-chelating bpym ligand to form a dinuclear Mn^II^ complex. Each Mn^II^ atom is six-coordinated in a considerably distorted octahedral environment defined by two N atoms of the bridging bpym ligand, and four O atoms from one sulfato anionic ligand and three water molecules. However, in the previously reported crystal structure of the analogous dinuclear cationic complex [Mn_2_(H_2_O)_8_(bpym)](SO_4_)_2_.2H_2_O, its single crystals were obtained from a water solution at 50 °C, each Mn^II^ atom is coordinated by two N atoms from bpym ligand and four O atoms from four water molecules (Ha, 2011).

The main contributions to the distortion of the octahedron are the tight N—Mn—N chelate angles [71.50 (8) and 71.46 (8)°] and the bulky SO_4_ groups, which results in non-linear *trans* axes [<N1—Mn1—O2 = 157.90 (9)° and <N3—Mn2—O9 = 155.79 (9)°], whereas the apical O1—Mn1—O3 and O8—Mn2—O10 bonds are roughly linear with the bond angles of 175.80 (9)° and 176.15 (8)°, respectively. In the two complexes, however, the apical N—Mn—O(SO_4_) bond angles are fairly different with <N2—Mn1—O4 = 178.47 (8)° and <N4—Mn2—O11 = 160.35 (8)°, because the coordination modes of the SO_4_ anions are somewhat different. Atom O4 in the complex with atom Mn1 occupies the equatorial position, but atom O11 in the other complex is inclined considerably to the equatorial plane. The Mn—N and Mn—O bond lengths are roughly equivalent, respectively (Table 1). The geometry of the SO_4_ ligands are nearly tetrahedral with the O—S—O bond angles of 107.83 (13)–111.48 (16)°, and the S—O bond distances are almost equal with 1.437 (2)–1.477 (2) Å. In the crystal structure, the complexes are linked by O—H···O hydrogen bonds between the water and sulfato ligands, forming a three-dimensional network (Fig. 2, Table 2). In addition, the complexes display numerous intermolecular π–π interactions between adjacent pyrimidine rings, the shortest ring centroid-centroid distance being 3.704 (2) Å.

### Experimental

MnSO_4_.H_2_O (0.1688 g, 0.999 mmol) and 2,2'-bipyrimidine (0.1587 g, 1.003 mmol) in H_2_O (20 ml) were refluxed for 1 h. After evaporation of the solvent, the residue was washed with ether and dried at 50 °C, to give a light yellow powder (0.3152 g) (Ha, 2011). Crystals suitable for X-ray analysis were obtained by slow evaporation from a mixture of water and dimethyl sulfoxide (DMSO) at 90 °C.

### Refinement

Carbon-bound H atoms were positioned geometrically and allowed to ride on their respective parent atoms [C—H = 0.95 Å and *U*_iso_(H) = 1.2*U*_eq_(C)]. The H atoms of the water ligands were located in a difference Fourier map then allowed to ride on their parent O atoms in the final cycles of refinement, with O—H = 0.84 Å and *U*_iso_(H) = 1.5 *U*_eq_(O). The highest peak (0.75 e Å^-3^) and the deepest hole (-0.62 e Å^-3^) in the difference Fourier map are located 0.86 Å and 0.72 Å from the atoms O14 and Mn2, respectively.

### Crystal data

**Table:**

[Mn_2_(SO_4_)_2_(C_8_H_6_N_4_)(H_2_O)_6_]	*F*(000) = 1152
*M**_r_* = 568.26	*D*_x_ = 1.994 Mg m^−^^3^
Monoclinic, *P*2_1_/*n*	Mo *K*α radiation, λ = 0.71073 Å
Hall symbol: -P 2yn	Cell parameters from 4745 reflections
*a* = 12.4401 (18) Å	θ = 2.4–28.3°
*b* = 13.2640 (19) Å	µ = 1.64 mm^−^^1^
*c* = 12.8951 (18) Å	*T* = 200 K
β = 117.199 (3)°	Block, pale yellow
*V* = 1892.5 (5) Å^3^	0.33 × 0.23 × 0.20 mm
*Z* = 4	

### Data collection

**Table:**

Bruker SMART 1000 CCD diffractometer	4652 independent reflections
Radiation source: fine-focus sealed tube	3069 reflections with *I* > 2σ(*I*)
graphite	*R*_int_ = 0.042
φ and ω scans	θ_max_ = 28.3°, θ_min_ = 1.9°
Absorption correction: multi-scan (*SADABS*; Bruker, 2000)	*h* = −16→14
*T*_min_ = 0.505, *T*_max_ = 0.721	*k* = −13→17
13624 measured reflections	*l* = −17→17

### Refinement

**Table:**

Refinement on *F*^2^	Secondary atom site location: difference Fourier map
Least-squares matrix: full	Hydrogen site location: inferred from neighbouring sites
*R*[*F*^2^ > 2σ(*F*^2^)] = 0.038	H-atom parameters constrained
*wR*(*F*^2^) = 0.110	*w* = 1/[σ^2^(*F*_o_^2^) + (0.0451*P*)^2^] where *P* = (*F*_o_^2^ + 2*F*_c_^2^)/3
*S* = 1.08	(Δ/σ)_max_ < 0.001
4652 reflections	Δρ_max_ = 0.75 e Å^−^^3^
272 parameters	Δρ_min_ = −0.62 e Å^−^^3^
0 restraints	Extinction correction: *SHELXL*, Fc^*^=kFc[1+0.001xFc^2^λ^3^/sin(2θ)]^-1/4^
Primary atom site location: structure-invariant direct methods	Extinction coefficient: 0.0063 (5)

### Special details

**Table:**

Geometry. All e.s.d.'s (except the e.s.d. in the dihedral angle between two l.s. planes) are estimated using the full covariance matrix. The cell e.s.d.'s are taken into account individually in the estimation of e.s.d.'s in distances, angles and torsion angles; correlations between e.s.d.'s in cell parameters are only used when they are defined by crystal symmetry. An approximate (isotropic) treatment of cell e.s.d.'s is used for estimating e.s.d.'s involving l.s. planes.
Refinement. Refinement of *F*^2^ against ALL reflections. The weighted *R*-factor *wR* and goodness of fit *S* are based on *F*^2^, conventional *R*-factors *R* are based on *F*, with *F* set to zero for negative *F*^2^. The threshold expression of *F*^2^ > σ(*F*^2^) is used only for calculating *R*-factors(gt) *etc*. and is not relevant to the choice of reflections for refinement. *R*-factors based on *F*^2^ are statistically about twice as large as those based on *F*, and *R*- factors based on ALL data will be even larger.

### Fractional atomic coordinates and isotropic or equivalent isotropic displacement parameters (Å^2^)

**Table:**

	*x*	*y*	*z*	*U*_iso_*/*U*_eq_	
Mn1	0.26775 (4)	0.05409 (3)	0.60729 (4)	0.01696 (14)	
S1	0.43118 (6)	0.18859 (5)	0.50146 (6)	0.01596 (18)	
O1	0.28486 (19)	−0.08475 (16)	0.52677 (18)	0.0272 (5)	
H1A	0.3524	−0.1059	0.5363	0.041*	
H1B	0.2528	−0.1369	0.5374	0.041*	
O2	0.39400 (18)	0.00236 (15)	0.77569 (16)	0.0239 (5)	
H2A	0.4336	0.0382	0.8348	0.036*	
H2B	0.4169	−0.0568	0.7983	0.036*	
O3	0.24123 (19)	0.18829 (16)	0.69190 (18)	0.0307 (6)	
H3A	0.2758	0.2102	0.7606	0.046*	
H3B	0.1933	0.2374	0.6692	0.046*	
O4	0.4044 (2)	0.12401 (17)	0.57997 (19)	0.0335 (6)	
O5	0.31606 (19)	0.22742 (16)	0.40891 (18)	0.0271 (5)	
O6	0.49194 (19)	0.12919 (17)	0.44799 (18)	0.0319 (6)	
O7	0.5076 (2)	0.27128 (16)	0.56795 (18)	0.0310 (6)	
N1	0.0832 (2)	0.07677 (17)	0.44664 (19)	0.0175 (5)	
N2	0.1216 (2)	−0.02504 (17)	0.64137 (19)	0.0167 (5)	
C1	0.0618 (3)	0.1279 (2)	0.3487 (2)	0.0223 (7)	
H1	0.1259	0.1646	0.3456	0.027*	
C2	−0.0501 (3)	0.1283 (2)	0.2535 (3)	0.0232 (7)	
H2	−0.0643	0.1642	0.1847	0.028*	
C3	0.1404 (3)	−0.0753 (2)	0.7387 (2)	0.0205 (6)	
H3	0.2185	−0.0739	0.8038	0.025*	
C4	−0.0104 (2)	0.0281 (2)	0.4461 (2)	0.0138 (6)	
Mn2	0.27208 (4)	0.03864 (3)	0.10592 (4)	0.01670 (14)	
S2	0.43943 (6)	0.17926 (5)	0.01112 (6)	0.01673 (18)	
O8	0.28871 (19)	−0.10874 (15)	0.03961 (18)	0.0263 (5)	
H8A	0.3584	−0.1216	0.0483	0.039*	
H8B	0.2573	−0.1559	0.0599	0.039*	
O9	0.38962 (18)	−0.00536 (16)	0.28077 (16)	0.0247 (5)	
H9A	0.4206	0.0376	0.3343	0.037*	
H9B	0.4414	−0.0517	0.3015	0.037*	
O10	0.24556 (18)	0.18206 (15)	0.17427 (17)	0.0251 (5)	
H10A	0.2581	0.2050	0.2395	0.038*	
H10B	0.1723	0.1983	0.1458	0.038*	
O11	0.3665 (2)	0.09401 (16)	0.01777 (19)	0.0291 (5)	
O12	0.3684 (2)	0.26991 (19)	−0.0240 (2)	0.0554 (8)	
O13	0.4819 (2)	0.15436 (17)	−0.07476 (18)	0.0305 (6)	
O14	0.5442 (2)	0.19069 (18)	0.12602 (18)	0.0377 (6)	
N3	0.0900 (2)	0.06865 (17)	−0.05238 (19)	0.0161 (5)	
N4	0.1205 (2)	−0.03640 (17)	0.1383 (2)	0.0170 (5)	
C5	0.0733 (3)	0.1240 (2)	−0.1460 (2)	0.0204 (6)	
H5	0.1409	0.1552	−0.1483	0.025*	
C6	−0.0389 (3)	0.1363 (2)	−0.2376 (2)	0.0225 (7)	
H6	−0.0503	0.1753	−0.3037	0.027*	
C7	0.1351 (3)	−0.0905 (2)	0.2315 (2)	0.0208 (7)	
H7	0.2137	−0.0973	0.2949	0.025*	
C8	−0.0082 (3)	0.0289 (2)	−0.0528 (2)	0.0146 (6)	

### Atomic displacement parameters (Å^2^)

**Table:**

	*U*^11^	*U*^22^	*U*^33^	*U*^12^	*U*^13^	*U*^23^
Mn1	0.0130 (3)	0.0182 (3)	0.0180 (2)	−0.00137 (17)	0.0057 (2)	0.00144 (18)
S1	0.0154 (4)	0.0149 (4)	0.0181 (4)	−0.0013 (3)	0.0081 (3)	−0.0016 (3)
O1	0.0226 (12)	0.0219 (12)	0.0391 (13)	−0.0005 (9)	0.0158 (11)	−0.0065 (10)
O2	0.0209 (12)	0.0226 (12)	0.0184 (10)	0.0019 (9)	0.0005 (9)	−0.0004 (9)
O3	0.0274 (13)	0.0243 (13)	0.0301 (12)	0.0048 (9)	0.0044 (10)	−0.0059 (10)
O4	0.0257 (13)	0.0330 (14)	0.0454 (14)	0.0004 (10)	0.0194 (11)	0.0163 (11)
O5	0.0227 (13)	0.0285 (13)	0.0274 (12)	0.0052 (9)	0.0091 (10)	0.0014 (10)
O6	0.0221 (13)	0.0362 (14)	0.0367 (13)	0.0021 (10)	0.0130 (11)	−0.0127 (11)
O7	0.0291 (14)	0.0299 (13)	0.0341 (12)	−0.0108 (10)	0.0144 (11)	−0.0113 (10)
N1	0.0174 (13)	0.0167 (13)	0.0178 (12)	−0.0015 (10)	0.0076 (11)	0.0005 (10)
N2	0.0147 (13)	0.0183 (13)	0.0158 (12)	−0.0005 (9)	0.0058 (10)	0.0011 (10)
C1	0.0209 (17)	0.0226 (16)	0.0243 (16)	0.0012 (12)	0.0113 (14)	0.0040 (13)
C2	0.0277 (18)	0.0235 (17)	0.0195 (15)	0.0006 (13)	0.0118 (14)	0.0040 (13)
C3	0.0189 (16)	0.0230 (16)	0.0160 (14)	0.0007 (12)	0.0048 (12)	0.0018 (12)
C4	0.0126 (14)	0.0151 (14)	0.0145 (14)	−0.0007 (10)	0.0067 (11)	−0.0016 (11)
Mn2	0.0142 (3)	0.0182 (3)	0.0166 (2)	−0.00171 (17)	0.00615 (19)	−0.00021 (18)
S2	0.0169 (4)	0.0160 (4)	0.0193 (4)	0.0012 (3)	0.0100 (3)	0.0013 (3)
O8	0.0253 (13)	0.0193 (12)	0.0411 (13)	−0.0016 (9)	0.0213 (11)	−0.0029 (10)
O9	0.0182 (12)	0.0269 (12)	0.0195 (11)	0.0052 (9)	0.0004 (9)	−0.0004 (9)
O10	0.0206 (12)	0.0254 (12)	0.0248 (11)	0.0008 (9)	0.0064 (10)	−0.0066 (9)
O11	0.0297 (13)	0.0260 (13)	0.0358 (13)	−0.0068 (9)	0.0186 (11)	−0.0001 (10)
O12	0.0575 (19)	0.0369 (16)	0.090 (2)	0.0299 (13)	0.0495 (18)	0.0336 (15)
O13	0.0343 (14)	0.0358 (14)	0.0312 (12)	−0.0135 (10)	0.0236 (11)	−0.0124 (10)
O14	0.0369 (15)	0.0439 (16)	0.0240 (12)	−0.0175 (11)	0.0068 (11)	−0.0047 (11)
N3	0.0154 (13)	0.0156 (12)	0.0170 (12)	−0.0024 (9)	0.0070 (10)	−0.0001 (10)
N4	0.0153 (13)	0.0191 (13)	0.0162 (12)	0.0015 (9)	0.0070 (10)	0.0008 (10)
C5	0.0228 (17)	0.0199 (16)	0.0232 (15)	−0.0019 (12)	0.0145 (13)	0.0036 (13)
C6	0.0251 (18)	0.0240 (17)	0.0204 (15)	0.0040 (13)	0.0120 (14)	0.0087 (13)
C7	0.0224 (17)	0.0205 (16)	0.0165 (14)	0.0032 (12)	0.0062 (13)	0.0054 (12)
C8	0.0174 (15)	0.0116 (14)	0.0145 (14)	−0.0008 (10)	0.0071 (12)	−0.0015 (11)

### Geometric parameters (Å, °)

**Table:**

Mn1—O4	2.103 (2)	Mn2—O11	2.105 (2)
Mn1—O2	2.1295 (19)	Mn2—O9	2.1327 (19)
Mn1—O1	2.172 (2)	Mn2—O8	2.181 (2)
Mn1—O3	2.190 (2)	Mn2—O10	2.184 (2)
Mn1—N1	2.303 (2)	Mn2—N3	2.287 (2)
Mn1—N2	2.308 (2)	Mn2—N4	2.332 (2)
S1—O7	1.448 (2)	S2—O12	1.437 (2)
S1—O6	1.464 (2)	S2—O13	1.466 (2)
S1—O4	1.476 (2)	S2—O14	1.467 (2)
S1—O5	1.476 (2)	S2—O11	1.477 (2)
O1—H1A	0.8400	O8—H8A	0.8400
O1—H1B	0.8400	O8—H8B	0.8400
O2—H2A	0.8400	O9—H9A	0.8400
O2—H2B	0.8400	O9—H9B	0.8400
O3—H3A	0.8400	O10—H10A	0.8400
O3—H3B	0.8400	O10—H10B	0.8400
N1—C4	1.328 (3)	N3—C8	1.328 (3)
N1—C1	1.348 (4)	N3—C5	1.345 (3)
N2—C4^i^	1.327 (3)	N4—C8^ii^	1.330 (3)
N2—C3	1.344 (4)	N4—C7	1.339 (3)
C1—C2	1.372 (4)	C5—C6	1.365 (4)
C1—H1	0.9500	C5—H5	0.9500
C2—C3^i^	1.368 (4)	C6—C7^ii^	1.376 (4)
C2—H2	0.9500	C6—H6	0.9500
C3—H3	0.9500	C7—H7	0.9500
C4—C4^i^	1.492 (5)	C8—C8^ii^	1.494 (5)
			
O4—Mn1—O2	92.12 (8)	O11—Mn2—O9	112.59 (9)
O4—Mn1—O1	91.74 (9)	O11—Mn2—O8	85.68 (8)
O2—Mn1—O1	91.52 (8)	O9—Mn2—O8	91.55 (8)
O4—Mn1—O3	92.43 (9)	O11—Mn2—O10	97.96 (8)
O2—Mn1—O3	87.86 (8)	O9—Mn2—O10	88.21 (8)
O1—Mn1—O3	175.80 (9)	O8—Mn2—O10	176.15 (8)
O4—Mn1—N1	109.97 (9)	O11—Mn2—N3	91.54 (9)
O2—Mn1—N1	157.90 (9)	O9—Mn2—N3	155.79 (9)
O1—Mn1—N1	87.48 (8)	O8—Mn2—N3	92.22 (8)
O3—Mn1—N1	91.53 (8)	O10—Mn2—N3	86.44 (8)
O4—Mn1—N2	178.47 (8)	O11—Mn2—N4	160.35 (8)
O2—Mn1—N2	86.41 (8)	O9—Mn2—N4	85.05 (8)
O1—Mn1—N2	87.90 (8)	O8—Mn2—N4	85.24 (8)
O3—Mn1—N2	87.92 (9)	O10—Mn2—N4	90.92 (8)
N1—Mn1—N2	71.50 (8)	N3—Mn2—N4	71.46 (8)
O7—S1—O6	110.25 (13)	O12—S2—O13	109.38 (15)
O7—S1—O4	109.04 (13)	O12—S2—O14	111.48 (16)
O6—S1—O4	109.73 (14)	O13—S2—O14	109.04 (14)
O7—S1—O5	110.26 (13)	O12—S2—O11	110.72 (15)
O6—S1—O5	109.03 (12)	O13—S2—O11	108.32 (13)
O4—S1—O5	108.51 (13)	O14—S2—O11	107.83 (13)
Mn1—O1—H1A	121.6	Mn2—O8—H8A	114.0
Mn1—O1—H1B	117.5	Mn2—O8—H8B	113.8
H1A—O1—H1B	102.6	H8A—O8—H8B	114.0
Mn1—O2—H2A	126.6	Mn2—O9—H9A	121.2
Mn1—O2—H2B	129.0	Mn2—O9—H9B	126.3
H2A—O2—H2B	104.4	H9A—O9—H9B	103.7
Mn1—O3—H3A	133.3	Mn2—O10—H10A	136.6
Mn1—O3—H3B	134.5	Mn2—O10—H10B	112.3
H3A—O3—H3B	92.2	H10A—O10—H10B	90.8
S1—O4—Mn1	145.49 (14)	S2—O11—Mn2	143.88 (14)
C4—N1—C1	116.2 (2)	C8—N3—C5	116.6 (2)
C4—N1—Mn1	116.88 (18)	C8—N3—Mn2	117.95 (18)
C1—N1—Mn1	126.6 (2)	C5—N3—Mn2	125.42 (19)
C4^i^—N2—C3	116.6 (2)	C8^ii^—N4—C7	116.3 (3)
C4^i^—N2—Mn1	117.25 (18)	C8^ii^—N4—Mn2	116.68 (18)
C3—N2—Mn1	125.96 (19)	C7—N4—Mn2	126.9 (2)
N1—C1—C2	121.7 (3)	N3—C5—C6	121.3 (3)
N1—C1—H1	119.2	N3—C5—H5	119.3
C2—C1—H1	119.2	C6—C5—H5	119.3
C3^i^—C2—C1	117.7 (3)	C5—C6—C7^ii^	118.0 (3)
C3^i^—C2—H2	121.2	C5—C6—H6	121.0
C1—C2—H2	121.2	C7^ii^—C6—H6	121.0
N2—C3—C2^i^	121.7 (3)	N4—C7—C6^ii^	121.6 (3)
N2—C3—H3	119.2	N4—C7—H7	119.2
C2^i^—C3—H3	119.2	C6^ii^—C7—H7	119.2
N2^i^—C4—N1	126.2 (2)	N3—C8—N4^ii^	126.1 (2)
N2^i^—C4—C4^i^	116.5 (3)	N3—C8—C8^ii^	117.3 (3)
N1—C4—C4^i^	117.4 (3)	N4^ii^—C8—C8^ii^	116.6 (3)
			
O7—S1—O4—Mn1	127.8 (3)	O14—S2—O11—Mn2	57.7 (3)
O6—S1—O4—Mn1	−111.4 (3)	O9—Mn2—O11—S2	−70.5 (3)
O5—S1—O4—Mn1	7.7 (3)	O8—Mn2—O11—S2	−160.4 (2)
O2—Mn1—O4—S1	−174.7 (3)	O10—Mn2—O11—S2	20.9 (2)
O1—Mn1—O4—S1	93.7 (3)	N3—Mn2—O11—S2	107.5 (2)
O3—Mn1—O4—S1	−86.7 (3)	N4—Mn2—O11—S2	137.0 (2)
N1—Mn1—O4—S1	5.8 (3)	O11—Mn2—N3—C8	167.6 (2)
O4—Mn1—N1—C4	173.38 (19)	O9—Mn2—N3—C8	−16.9 (3)
O2—Mn1—N1—C4	−5.4 (4)	O8—Mn2—N3—C8	81.9 (2)
O1—Mn1—N1—C4	82.4 (2)	O10—Mn2—N3—C8	−94.5 (2)
O3—Mn1—N1—C4	−93.5 (2)	N4—Mn2—N3—C8	−2.31 (19)
N2—Mn1—N1—C4	−6.18 (19)	O11—Mn2—N3—C5	−12.6 (2)
O4—Mn1—N1—C1	−0.9 (3)	O9—Mn2—N3—C5	162.9 (2)
O2—Mn1—N1—C1	−179.7 (2)	O8—Mn2—N3—C5	−98.4 (2)
O1—Mn1—N1—C1	−91.8 (2)	O10—Mn2—N3—C5	85.2 (2)
O3—Mn1—N1—C1	92.3 (2)	N4—Mn2—N3—C5	177.4 (2)
N2—Mn1—N1—C1	179.5 (3)	O11—Mn2—N4—C8^ii^	−29.3 (4)
O2—Mn1—N2—C4^i^	−173.7 (2)	O9—Mn2—N4—C8^ii^	176.0 (2)
O1—Mn1—N2—C4^i^	−82.0 (2)	O8—Mn2—N4—C8^ii^	−92.0 (2)
O3—Mn1—N2—C4^i^	98.4 (2)	O10—Mn2—N4—C8^ii^	87.9 (2)
N1—Mn1—N2—C4^i^	6.04 (19)	N3—Mn2—N4—C8^ii^	1.92 (19)
O2—Mn1—N2—C3	0.8 (2)	O11—Mn2—N4—C7	147.2 (3)
O1—Mn1—N2—C3	92.4 (2)	O9—Mn2—N4—C7	−7.5 (2)
O3—Mn1—N2—C3	−87.2 (2)	O8—Mn2—N4—C7	84.5 (2)
N1—Mn1—N2—C3	−179.5 (2)	O10—Mn2—N4—C7	−95.6 (2)
C4—N1—C1—C2	−0.9 (4)	N3—Mn2—N4—C7	178.5 (2)
Mn1—N1—C1—C2	173.5 (2)	C8—N3—C5—C6	−1.5 (4)
N1—C1—C2—C3^i^	0.4 (4)	Mn2—N3—C5—C6	178.8 (2)
C4^i^—N2—C3—C2^i^	1.1 (4)	N3—C5—C6—C7^ii^	0.2 (4)
Mn1—N2—C3—C2^i^	−173.4 (2)	C8^ii^—N4—C7—C6^ii^	0.6 (4)
C1—N1—C4—N2^i^	0.3 (4)	Mn2—N4—C7—C6^ii^	−176.0 (2)
Mn1—N1—C4—N2^i^	−174.6 (2)	C5—N3—C8—N4^ii^	2.0 (4)
C1—N1—C4—C4^i^	−179.3 (3)	Mn2—N3—C8—N4^ii^	−178.3 (2)
Mn1—N1—C4—C4^i^	5.8 (4)	C5—N3—C8—C8^ii^	−177.3 (3)
O12—S2—O11—Mn2	−64.5 (3)	Mn2—N3—C8—C8^ii^	2.4 (4)
O13—S2—O11—Mn2	175.6 (2)		

Symmetry codes: (i) −*x*, −*y*, −*z*+1; (ii) −*x*, −*y*, −*z*.

### Hydrogen-bond geometry (Å, °)

**Table:**

*D*—H···*A*	*D*—H	H···*A*	*D*···*A*	*D*—H···*A*
O1—H1A···O6^iii^	0.84	1.88	2.709 (3)	170.
O1—H1B···O12^iv^	0.84	1.90	2.700 (3)	160.
O2—H2A···O13^v^	0.84	1.86	2.655 (3)	158.
O2—H2B···O14^iii^	0.84	1.98	2.804 (3)	168.
O3—H3A···O12^v^	0.84	2.60	3.434 (4)	175.
O3—H3B···O14^vi^	0.84	1.93	2.721 (3)	157.
O8—H8A···O13^vii^	0.84	1.91	2.745 (3)	177.
O8—H8B···O5^iv^	0.84	1.93	2.766 (3)	173.
O9—H9A···O6	0.84	1.80	2.636 (3)	178.
O9—H9B···O4^iii^	0.84	2.06	2.839 (3)	153.
O10—H10A···O5	0.84	1.98	2.804 (3)	165.
O10—H10B···O7^viii^	0.84	1.87	2.705 (3)	174.

Symmetry codes: (iii) −*x*+1, −*y*, −*z*+1; (iv) −*x*+1/2, *y*−1/2, −*z*+1/2; (v) *x*, *y*, *z*+1; (vi) *x*−1/2, −*y*+1/2, *z*+1/2; (vii) −*x*+1, −*y*, −*z*; (viii) *x*−1/2, −*y*+1/2, *z*−1/2.
